# Agrimol B alleviates cisplatin-induced acute kidney injury by activating the Sirt1/Nrf2 signaling pathway in mice: Agrimol B alleviates cisplatin-induced acute kidney injury

**DOI:** 10.3724/abbs.2023285

**Published:** 2024-02-23

**Authors:** Jiarui Tang, Longhui Li, Zhijian Chen, Cuiting Liao, Kai Hu, Yongqiang Yang, Jiayi Huang, Li Tang, Li Zhang, Longjiang Li

**Affiliations:** 1 Department of Pathophysiology School of Basic Medical Sciences Chongqing Medical University Chongqing 400016 China; 2 Laboratory of Stem Cell and Tissue Engineering Chongqing Medical University Chongqing 400016 China; 3 Department of Health Management Centre Chongqing General Hospital Chongqing 401147 China; 4 Department of Pathophysiology Shihezi University School of Medicine Shihezi 832000 China

**Keywords:** cisplatin, acute kidney injury, agrimol B, Sirt1, oxidative stress

## Abstract

Cisplatin (CDDP) is a widely used chemotherapeutic agent that has remarkable antineoplastic effects. However, CDDP can cause severe acute kidney injury (AKI), which limits its clinical application. Agrimol B is the main active ingredient found in *Agrimonia pilosa* Ledeb and has a variety of pharmacological activities. The effect of agrimol B on CDDP-induced renal toxicity has not been determined. To investigate whether agrimol B has a protective effect against CDDP-induced AKI, we first identify Sirtuin 1 (Sirt1) as a critical target protein of agrimol B in regulating AKI through network pharmacology analysis. Subsequently, the AKI mouse model is induced by administering a single dose of CDDP via intraperitoneal injection. By detecting the serum urea nitrogen and creatinine levels, as well as the histopathological changes, we confirm that agrimol B effectively reduces CDDP-induced AKI. In addition, treatment with agrimol B counteracts the increase in renal malondialdehyde level and the decrease in superoxide dismutase (SOD), catalase and glutathione levels induced by CDDP. Moreover, western blot results reveal that agrimol B upregulates the expressions of Sirt1, SOD2, nuclear factor erythroid2-related factor 2, and downstream molecules, including heme oxygenase 1 and NAD(P)H quinone dehydrogenase 1. However, administration of the Sirt1 inhibitor EX527 abolishes the effects of agrimol B. Finally, we establish a tumor-bearing mouse model and find that agrimol B has a synergistic antitumor effect with CDDP. Overall, agrimol B attenuates CDDP-induced AKI by activating the Sirt1/Nrf2 signaling pathway to counteract oxidative stress, suggesting that this compound is a potential therapeutic agent for the treatment of CDDP-induced AKI.

## Introduction

Cisplatin (CDDP) is a platinum-based chemotherapeutic agent that has been widely used to treat various malignant solid tumors, including testicular cancer, ovarian cancer, non-small cell lung cancer, cervical cancer, head and neck cancer, bladder cancer and gastric cancer [ 1, 2]. Despite its potent antineoplastic effects, CDDP also has a variety of side effects, especially nephrotoxicity, which is the principal factor limiting its use and efficacy [3]. Approximately 25%‒30% of patients treated with CDDP progress to acute kidney injury (AKI), which is characterized by a rapid loss of kidney function and is associated with high morbidity and mortality [4]. The incidence of chronic kidney disease is also significantly increased in patients who survive AKI induced by CDDP [5]. At present, there is still no effective method to prevent or treat CDDP-induced AKI.

The mechanism of CDDP-induced AKI is very complex, and a lot of studies have shown that oxidative stress, inflammation, apoptosis, and DNA damage are involved in its pathogenesis [ 3, 6]. In particular, CDDP is hydrolyzed within proximal tubule cells, and its products bind to intracellular antioxidants, resulting in the depletion and inactivation of antioxidants. This, in turn, leads to the abnormal accumulation of reactive oxygen species (ROS), ultimately damaging cellular structures and various components, such as DNA, proteins, and cell membranes [ 3, 5]. The production of ROS causes the decrease of renal blood flow and tubular lesions. Additionally, it induces cell necrosis by promoting lipid peroxidation of cell membranes and DNA damage [7]. Therefore, alleviating oxidative stress is considered a potential strategy to improve CDDP-induced AKI.

Based on preclinical findings, the use of antioxidant components in natural products appears to be a promising approach for preventing CDDP-induced AKI without compromising antitumor activity [8]. The traditional Chinese medicine *Agrimonia pilosa* Ledeb, listed in the Pharmacopoeia of the People’s Republic of China, has antineoplastic, antioxidative, anti-inflammatory, and antihyperglycemic effects [ 9‒ 11]. Agrimol B, one of the main active ingredients, has also been shown to mitigate oxidative stress and counteract various types of cancers [11]. However, it remains unclear whether agrimol B has the potential to protect against CDDP-induced AKI.

In the present study, we investigated the impact and mechanism of action of agrimol B in CDDP-induced AKI. Our results showed that agrimol B could ameliorate oxidative stress and protect against CDDP-induced AKI through the Sirt1/Nrf2 signaling pathway while also exhibiting a synergistic antitumor effect with CDDP. Thus, this study revealed the potential target and possible mechanism of action of agrimol B in the treatment of CDDP-induced AKI.

## Materials and Methods

### Network pharmacology analysis

In this study, information on *Agrimonia pilosa* Ledeb was obtained from the literature and from the Traditional Chinese Medicine Systems Pharmacology (TCMSP) database ( https://tcmspe.com/tcmsp.php). An oral bioavailability (OB) ≥ 30%, a drug similarity (DL) ≥0.18 and a blood‒brain barrier (BBB) ≥ 0.3 were used as screening criteria to identify the characteristic components of agrimol B. The chemical structure of agrimol B was determined using PubChem ( https://pubchem.ncbi.nlm.nih.gov/). The target prediction database Swiss Target Forecast ( http://swisstargetprediction.ch/) was subsequently used to predict the possible targets. The chemical structure of the target compound was uploaded to the UniProt database ( https://www.uniprot.org/), and the species “acute kidney injury” was selected. The related targets of agrimol B in AKI were identified. AKI-related genes were obtained from the DisGeNet database ( https://www.disgenet.org/), Online Mendelian Inheritance in Man (OMIM) ( https://www.omim.org/), and GeneCard database ( https://www.genecards.org/) to identify the genes associated with the target disease. The keyword “acute kidney injury” was used for the search.

Furthermore, all AKI-related gene expression data were obtained by combining the aforementioned results. The Venn diagram website ( http://bioinformatics.psb.ugent.be/webtools/VENN/) was used to create a Venn diagram illustrating the potential targets of agrimol B and the disease targets of AKI. The STRING database can provide information on protein-protein interactions (PPIs) and their levels of interaction based on confidence scores. The AKI targets regulated by agrimol B were input into String ( https://string-db.org/) to obtain relevant information about the PPIs. After the potential targets were imported into Cytoscape v3.6.1 software, the PPI network was visualized, and the core targets with a degree value higher than the average node were obtained. The OmicShare platform ( https://www.omicshare.com/) was used for Gene Ontology (GO) and Kyoto Encyclopedia of Genes and Genomes (KEGG) pathway enrichment analysis, and the results were sorted based on an adjusted *P* value ≤ 0.05. The top 20 enriched pathways for both GO and KEGG were analyzed.

### Reagents

Cisplatin and EX527 were purchased from MedChemExpress (Shanghai, China). Agrimol B was obtained from Shanghai Yuanye Bio-Technology Co., Ltd. (Shanghai, China). Dimethyl sulfoxide (DMSO), blood urea nitrogen (BUN), malondialdehyde (MDA), superoxide dismutase (SOD), catalase (CAT), and glutathione (GSH) assay kits were purchased from Nanjing Jiancheng Bioengineering Institute (Nanjing, China). An assay kit for determining the serum creatinine (Scr) concentration was obtained from ZCIBIO Technology Co., Ltd. (Shanghai, China). RIPA lysis buffer, protease inhibitor cocktail, phosphatase inhibitor cocktail and SDS-PAGE sample loading buffer were obtained from Beyotime Biotechnology (Shanghai, China). PEG400 was obtained from Sigma-Aldrich (St Louis, USA). A bicinchoninic acid (BCA) protein assay kit was obtained from Pierce Biotechnology (Rockford, USA). Enhanced chemiluminescence reagent was purchased from Biosharp (Beijing, China). The FastPure Cell/Tissue Total RNA Isolation kit, HiScript II Q RT SuperMix for qPCR (+gDNA wiper) and ChamQ SYBR qPCR Master Mix were purchased from Vazyme Biotech (Nanjing, China). Antibodies against SOD2 and β-actin were purchased from Proteintech (Wuhan, China). The antibody against Sirtuin 1 (Sirt1) was purchased from Wanlei Life Sciences Co., Ltd. (Shenyang, China). Antibodies against nuclear factor erythroid 2-related factor 2 (Nrf2) and NAD(P)H quinone dehydrogenase 1 (NQO-1) were purchased from Abcam (Cambridge, USA). The antibody against heme oxygenase 1 (HO-1) was purchased from Abmart Shanghai Co., Ltd. (Shanghai, China). The secondary anti-mouse/rabbit antibodies were purchased from Cell Signaling Technology (Beverly, USA).

### Animals and experimental design

BALB/c male mice (aged 6‒8 weeks, weighing 18‒22 g) were purchased from Byrness Weil Biotech Ltd. (Chongqing, China). The mice were housed under standard laboratory conditions, which included a 12/12 h light/dark cycle, an environment of 23±2°C, a relative humidity of 50%–60%, and free access to standard rodent chow and water, unless otherwise specified. The mice were acclimatized to the laboratory conditions two days before the experiment. The experimental procedures were approved by the Research Ethics Committee of Chongqing Medical University (No. IACUC-CQMU-2023-0353).

In the first set of experiments, the mice were randomly assigned into four groups. Based on the estimation of degrees of freedom from ANOVA [12], we selected six mice per group for the study. The groups were as follows ( *n*=6): control group, agrimol B group, CDDP group, and CDDP+agrimol B group. Nephrotoxicity was induced by intraperitoneal injection of CDDP (20 mg/kg) dissolved in PEG400. In the CDDP+agrimol B group, agrimol B (5 mg/kg) dissolved in a mixture of DMSO and corn oil (v:v=1:9) was intraperitoneally administered to the mice after 1 h, 12 h and 24 h of CDDP exposure, respectively. In parallel with the CDDP+agrimol B group, agrimol B was administered to the mice at the same time points after the intraperitoneal injection of PEG400. Mice in the control group were treated with vehicle at the corresponding time points. The vehicle for CDDP consisted of the solvent PEG400, while for agrimol B, it was composed of DMSO and corn oil at a ratio of 1:9 (v:v).

To explore the potential mechanism by which agrimol B affects CDDP-induced AKI, the specific inhibitor EX527 was applied to inhibit Sirt1. EX527 (10 mg/kg) dissolved in DMSO or corn oil (v:v=1:49) was intraperitoneally injected 12 h or 1 h before CDDP injection, respectively. Mice in the control group were treated with vehicle at the corresponding time points.

Thirty-six hours after CDDP injection, the mice were anaesthetized and sacrificed. Blood was collected from the retroorbital venous plexus for biochemical examination, and kidney tissue was obtained for biochemical and pathological assessment.

### Renal function evaluation

Renal function was evaluated by measuring Scr and BUN levels. The blood samples were coagulated at room temperature and then centrifuged at 5000 *g* and 4°C for 10 min to collect the serum. The serum levels of Scr and BUN were determined using the corresponding kits according to the manufacturer’s instructions.

### Histopathological observation of the kidney

The kidney samples were fixed in 4% paraformaldehyde. Then, the samples were dehydrated using 100% ethanol, infiltrated with soft paraffin, and embedded in molten paraffin according to the standard procedure. The paraffin sections were sliced into 5-μm thick sections.

For hematoxylin and eosin (H&E) staining, the slides were placed first in xylene to clear the paraffin. Subsequently, the samples were hydrated in 100% ethanol, 95% ethanol, 70% ethanol, and running tap water at room temperature. Then, the samples were stained in the hematoxylin and eosin solution. Finally, the slides were dipped in 95% ethanol, 100% ethanol, and xylene for dehydration step by step.

For periodic acid-schiff (PAS) staining, the protocol of deparaffinization and rehydration is the same with H&E staining. Then, the slides were firstly placed in the amylase solution and rinsed with distilled water (dH _2_O). Secondly, the samples were dipped in 0.5% periodic acid and washed with dH _2_O. Next, the slides were stained in the Schiff`s reagent and washed with running lukewarm tap water in order to develop a pink color. Subsequently, the samples were counterstained in the hematoxylin. Finally, the slides were dehydrated.

Both the H&E and PAS staining sections were observed under a microscope (Olympus, Tokyo, Japan). The degree of damage in the renal tubules was classified into five grades: normal kidney, <25% injury, 25%–50% injury, 50%–75% injury and >75% injury. These grades are assigned scores ranging from 0 to 4 [13]. The tissue sections were scored in a blinded manner.

### Oxidative stress evaluation

Lipid peroxidation was quantified by measuring the content of MDA. Oxidative stress defense was determined by SOD, CAT and GSH levels. All procedures were performed according to the kits’ instructions.

### Western blot analysis

Proteins were extracted from frozen kidney tissue using RIPA lysis buffer supplemented with a 1:100 protease inhibitor cocktail for general use, after which a 1:100 phosphatase inhibitor cocktail was added. The protein concentration was determined using a BCA protein assay kit. The protein samples were mixed with 5× loading buffer at a ratio of 1:4 and boiled for 10 min at 100°C. According to the protein content, samples with equal amounts of protein were separated by SDS-PAGE and transferred to nitrocellulose membranes (Millipore, Billerica, USA). Skim milk powder dissolved in TBST was used to block nonspecific binding sites. The membranes were subsequently incubated overnight at 4°C with primary antibodies against SOD2, Sirt1, Nrf2, NQO-1, HO-1, and β-actin. Afterward, the sections were incubated for 2 h with HRP-conjugated anti-mouse/rabbit IgG secondary antibodies. TBST was used to wash the membranes after incubation with primary and secondary antibodies. Enhanced chemiluminescence (ECL) kits were used to visualize the proteins on the membranes. Western blots were quantified using Image Lab (Bio-Rad, Hercules, USA).

### Real-time quantitative PCR

Total RNA was extracted from mouse kidneys using a FastPure cell/tissue total RNA isolation kit and then reverse-transcribed into cDNA with HiScript II Q RT SuperMix for qPCR. Real-time polymerase chain reaction (RT-PCR) was performed using ChamQ SYBR qPCR master mix in a real-time PCR detector (Bio-Rad). The expression levels of the target genes were calculated with the 2 ^–ΔΔCt^ method using *β-actin* as an internal control.

The sequences of the primers used are as follows: *Sirt1*, forward 5′-TGCCTGTTGAGGATTTGGTG-3′ and reverse 5′-TGACTTTCTGAGGTGTGAACTG-3′; *SOD2*, forward 5′-CAGACCTGCCTTACGACTATGG-3′ and reverse 5′-CTGAAGAGCGACCTGAGTTGTA-3′; *Nrf2*, forward 5′-CAGCACATCCAGACATT-3′ and reverse 5′-TCCAGGGCAAGCGACTCAT-3′; *HO-1*, forward 5′-CCAAAAGCACATCCAGCCAG-3′ and reverse 5′-CAGCAGTCGTGGTCAGTCAA-3′; *NQO-1*, forward 5′-CAGCCAATCAGCGTTCGGTA-3′ and reverse 5′-CTGGAAATGATGGGGTTGAAGT-3′; and *β-actin*, forward 5′-CATCCGTAAAGACCTCTATGCCAAC-3′ and reverse 5′-ATGGAGCCACCGATCCACA-3′.

### Tumor-bearing mouse model

A total of 5×10 ^6^ 4T1 breast cancer cells in 100 μL of PBS were subcutaneously injected into the right flank of the mice. Tumor growth was routinely monitored, and tumor volume (mm ^3^) was calculated as follows: volume=length×width ^2^×0.52. When the average tumor volume reached approximately 200 mm ^3^, the mice were randomly divided into three groups ( *n*=5): the control group, CDDP group, and CDDP+agrimol B group. The mice in the control group were treated with vehicle; the CDDP (5 mg/kg, i.p.) group received one dose of CDDP every four days. The CDDP+agrimol B group received agrimol B at 1 h after each CDDP injection, and the last CDDP injection was followed by agrimol B administration at 1 h, 12 h, and 24 h. Thirty-six hours after the last exposure to CDDP (total of 3 times), the mice were sacrificed, and the tumors were weighed, photographed and then fixed in 4% paraformaldehyde for pathological examination.

### Statistical analysis

The data were analyzed using GraphPad Prism 8.4.2 (GraphPad, San Diego, USA) and expressed as the mean±standard error of the mean (SEM) of three independent experiments. Student’s *t* test was used to compare the differences between two groups, and one-way ANOVA was used to analyze multiple groups. *P*<0.05 was considered significantly different.

## Results

### Network, functional enrichment analysis and target pathways of agrimol B in the treatment of AKI

**Figure 1:**
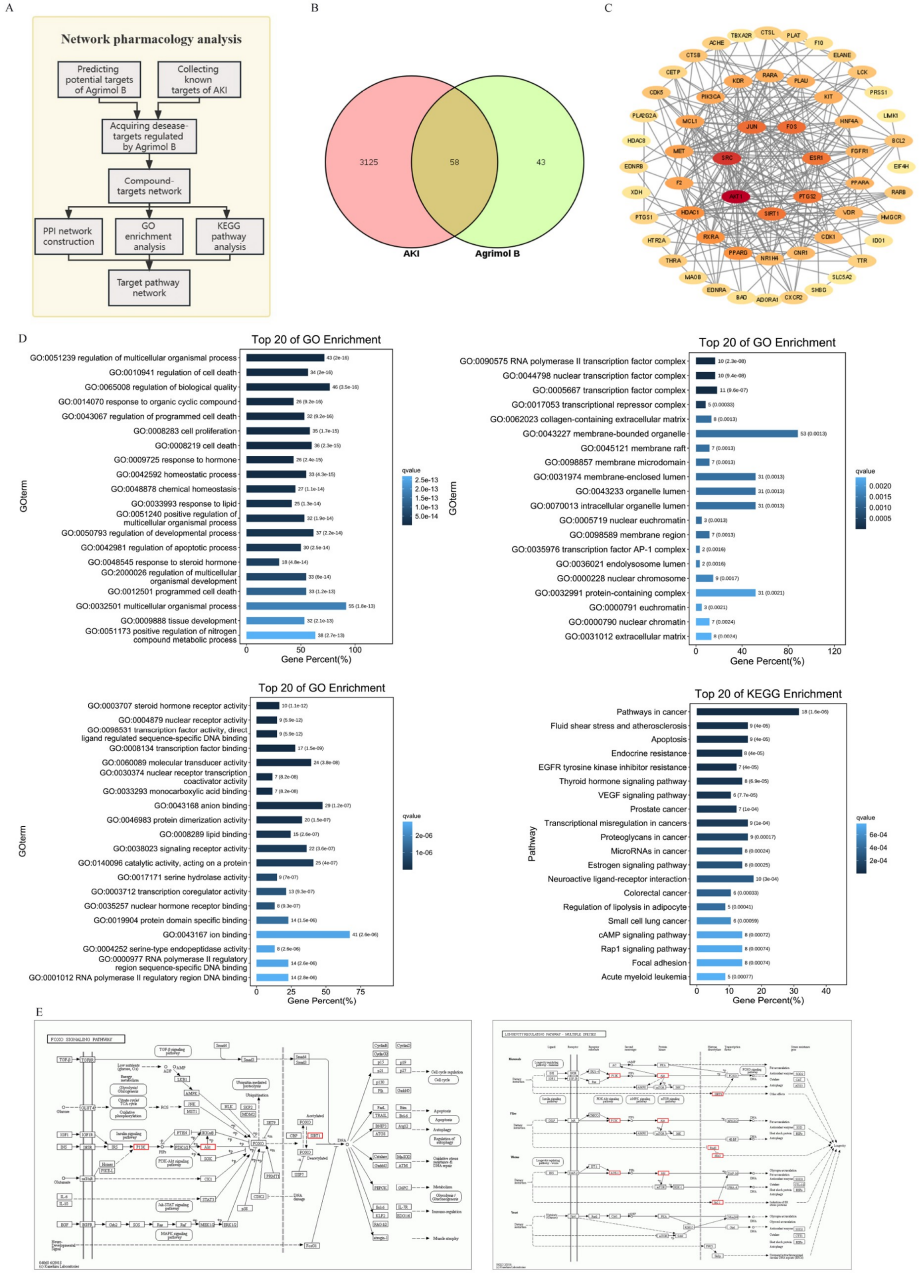
Figure 1 Network, functional enrichment analysis and target pathways of agrimol B against AKI (A) Flow chart of the network pharmacology analysis. (B) Venn diagram of common targets between AKI and agrimol B. (C) PPI network of proteins. (D) GO biological process, GO cellular component, GO molecular function and KEGG enrichment analyses. (E) FOXO signaling pathway and longevity-regulating pathway.

Based on the network pharmacology analysis flow chart ( Figure 1A), we initially gathered 101 potential targets of agrimol B using the TCMSP database. Additionally, we identified 3183 known targets of AKI by utilizing the OMIM, DisgeNet and GeneCards databases. A total of 58 agrimol B targets regulating AKI are shown in the Venn diagram ( Figure 1B).

To identify the common targets of agrimol B in AKI, we obtained protein-protein interaction data through network pharmacology and a PPI network. The results of the PPI analysis showed that the three critical target proteins of agrimol B-AKI were AKT1, SRC and Sirt1 ( Figure 1C).

To elucidate the biological processes associated with the core targets, we conducted GO analysis to determine the enrichment of genes in the biological process, cellular component and molecular function categories. The top 20 related functions are displayed in Figure 1D. According to the KEGG enrichment pathway analysis, 187 pathways were enriched. The first 20 KEGG pathways were screened using an enrichment factor and a *P* value less than 0.05 ( Figure 1D). According to the results of the GO and KEGG functional enrichment analyses, Sirt1 was identified as a crucial target of multiple signaling pathways involved in regulating oxidative stress in agrimol B-AKI patients, and these pathways included the FOXO signaling pathway and longevity regulating pathway ( Figure 1E).

Based on the results of network pharmacology screening, we hypothesized that agrimol B could protect against CDDP-induced AKI by targeting the protein Sirt1, and further investigations were conducted in animal experimental models to verify this hypothesis.

### Agrimol B protects the kidney against CDDP-induced AKI in mice

**Figure 2:**
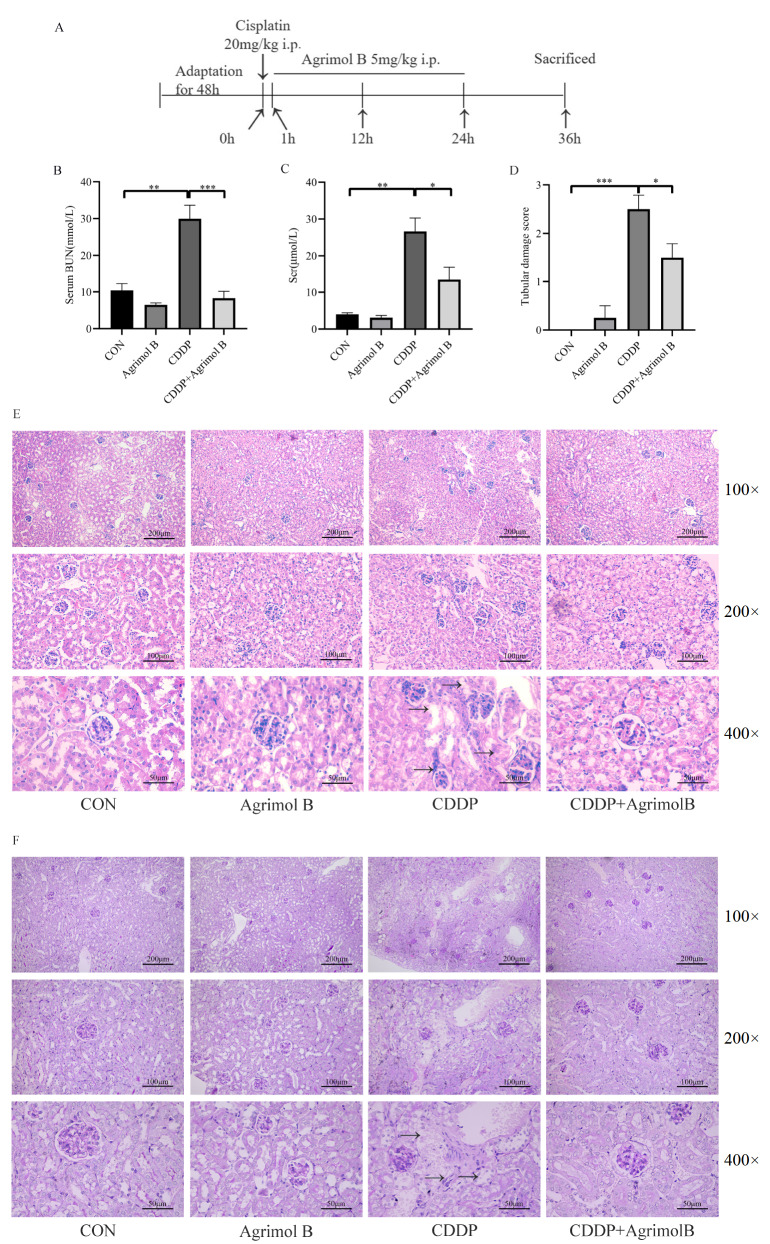
Figure 2 Agrimol B protected the kidney against CDDP-induced AKI (A) Experimental design. (B) The serum BUN level in each group (n=6 mice per group). (C) The Scr level in each group (n=6 mice per group). (D) Tubular damage score in each group (n=6 mice per group). (E) Kidneys stained with H&E. Representative histological sections were magnified (100×, 200× and 400×) and photographed. (F) Kidneys stained with PAS. Representative histological sections were magnified (100×, 200× and 400×) and photographed. SData are presented as the mean±SEM. *P<0.05, **P<0.01 and ***P<0.001.

Male BALB/c mice were injected once with CDDP (20 mg/kg, i.p.) to induce AKI, followed by treatment with agrimol B (5 mg/kg, i.p.) 3 times ( Figure 2A). Scr and BUN are the most commonly used markers of kidney function. Thirty-six hours after CDDP exposure, the BUN and Scr levels were significantly greater in the mice treated with CDDP than those in the control mice. Agrimol B administration decreased the Scr and BUN levels in CDDP-exposed mice. There was no significant difference in BUN or Scr level between the agrimol B group and the control group ( Figure 2B,C).

Microscopic examination via H&E and PAS staining revealed that the control group had normal tubular and glomerular structures. CDDP treatment caused severe pathological changes in the kidney, including glomerular structural damage, loss of the brush border, tubular dilation, vacuolization, and extensive detachment and necrosis of the tubular epithelium, as indicated by the black arrows. In contrast, treatment with agrimol B attenuated all of these impairments and protected kidney tissue from damage ( Figure 2E,F). The tubular damage score also indicated that agrimol B alleviated CDDP-induced histopathological damage ( Figure 2D). These data showed that agrimol B was protective against CDDP-induced AKI.

### Agrimol B diminishes CDDP-induced oxidative stress in mice

**Figure 3:**
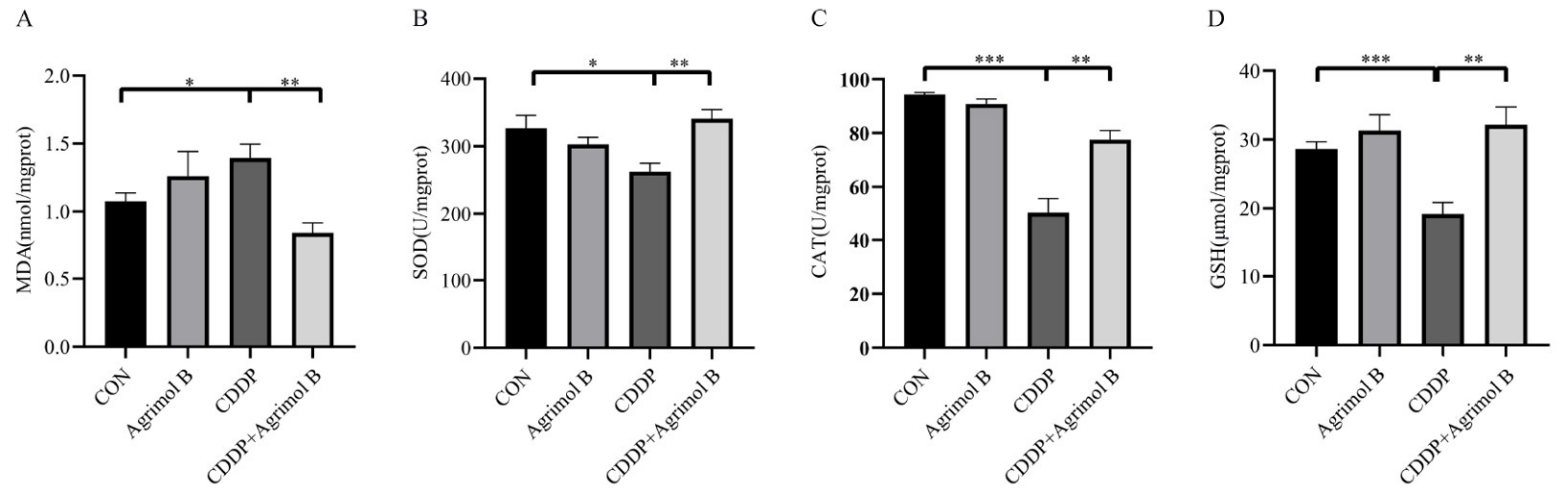
Figure 3 Agrimol B diminished CDDP-induced oxidative stress in mice (A) The MDA content in each group (n=6 mice per group). (B) SOD activity in each group (n=6 mice per group). (C) CAT activity in each group (n=6 mice per group). (D) GSH level in each group (n=6 mice per group). All the levels were determined by MDA, SOD, CAT and GSH assays after homogenization of the kidney tissue. Data are presented as the mean±SEM. *P<0.05, **P<0.01 and ***P<0.001.

Oxidative stress plays a crucial role in the pathogenesis of CDDP-induced AKI. MDA is the final product of lipid peroxidation and is generated due to the overproduction of free radicals. It is considered a marker of cell injury and cytotoxicity. Various molecules, including SOD, CAT and GSH, which play important roles in countering ROS, protect against oxidative stress in the body. We next determined whether treatment with agrimol B alters oxidative stress in the mouse model. Compared with the control group, the CDDP group showed a significant increase in MDA content and a marked decrease in SOD, CAT and GSH levels. In contrast, treatment with agrimol B notably reduced the MDA content and increased the SOD, CAT and GSH levels ( Figure 3). Our results indicated that agrimol B could protect the kidney from CDDP-induced oxidative stress.

### Agrimol B reverses CDDP-induced inhibition of Nrf2, HO-1 and NQO-1 expressions in mice

**Figure 4:**
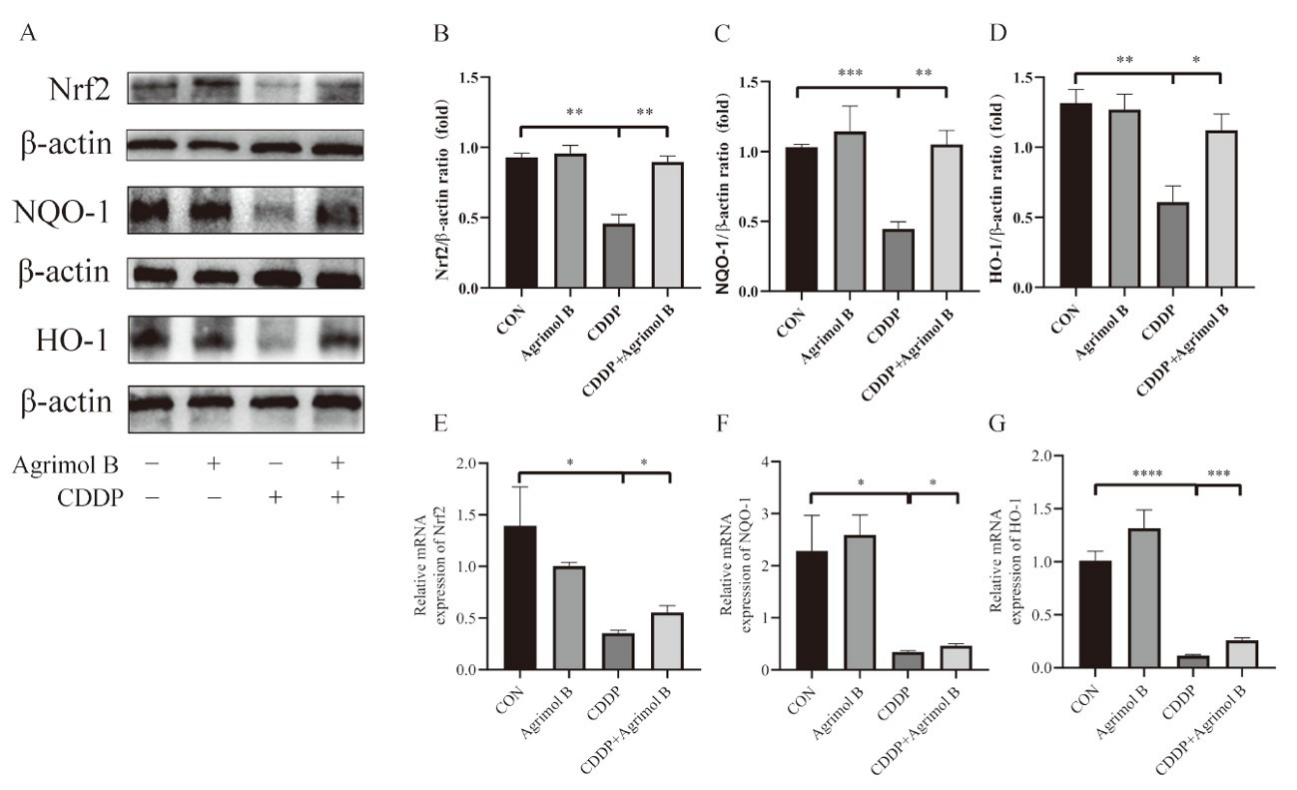
Figure 4 Agrimol B activated CDDP-induced inhibition of Nrf2, HO-1 and NQO-1 expression in mice (A) Protein expressions of Nrf2, HO-1 and NQO-1. (B) Densitometric analysis of Nrf2 protein expression normalized to that of β-actin. (C) Densitometric analysis of NQO-1 protein expression normalized to that of β-actin. (D) Densitometric analysis of HO-1 protein expression normalized to that of β-actin. (E) Relative mRNA expression of Nrf2. (F) Relative mRNA expression of NQO-1. (G) Relative mRNA expression of HO-1. Data are presented as the mean±SEM. *P<0.05, **P<0.01, ***P<0.001 and ****P<0.0001.

Furthermore, agrimol B attenuated CDDP-induced oxidative stress. Nrf2 is a crucial antioxidant transcription factor that interacts with its downstream antioxidant response elements, HO-1 and NQO-1, to maintain intracellular redox homeostasis. Therefore, we further explored whether agrimol B is involved in the expressions of molecules in the Nrf2 signaling pathway. We found that agrimol B could upregulate the CDDP-induced decreases in the Nrf2, HO-1 and NQO-1 protein expressions as well as their mRNA levels ( Figure 4), suggesting that agrimol B could protect against CDDP-induced oxidative stress by activating the Nrf2 signaling pathway.

### Agrimol B reverses CDDP-induced inhibitory effects on Sirt1 and SOD2 in mice

**Figure 5:**
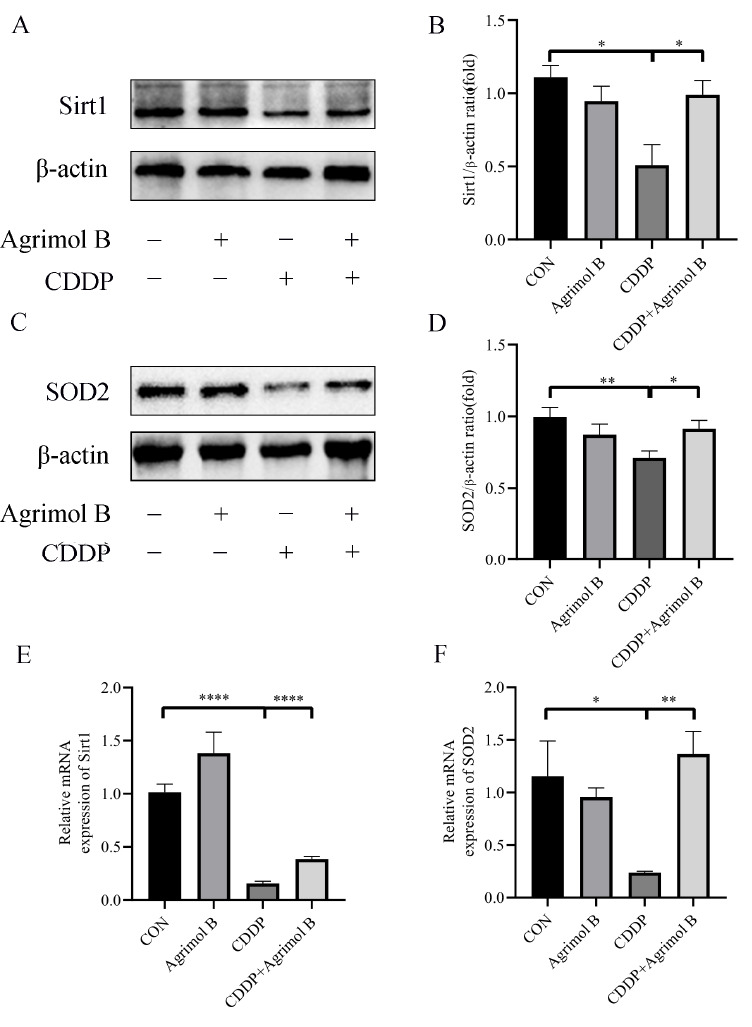
Figure 5 Agrimol B reversed the CDDP-induced inhibition of Sirt1 and SOD2 expressions in mice (A) Protein expression of Sirt1. (B) Densitometric analysis of Sirt1 protein expression normalized to that of β-actin. (C) Protein expression of SOD2. (D) Densitometric analysis of SOD2 protein expression normalized to that of β-actin. (E) Relative mRNA expression of Sirt1. (F) Relative mRNA expression of SOD2. Data are presented as the mean±SEM. *P<0.05, **P<0.01, and ****P<0.0001.

Sirt1, a histone deacetylase that regulates energy homeostasis, protects against oxidative stress, aging, and inflammation in organisms. Through network pharmacology analysis, agrimol B was predicted to be an important target of agrimol B in AKI. As shown in Figure 5A,B, agrimol B upregulated Sirt1 expression. The expression of SOD2, a crucial antioxidant enzyme, also showed the same trend as that of Sirt1 ( Figure 5C,D). RT-PCR results showed that CDDP similarly reduced the mRNA levels of *Sirt1* and *SOD2*, which were significantly increased after treatment with agrimol B ( Figure 5E,F).

### Sirt1 inhibition abrogates the protective effect of agrimol B on CDDP-induced AKI

**Figure 6:**
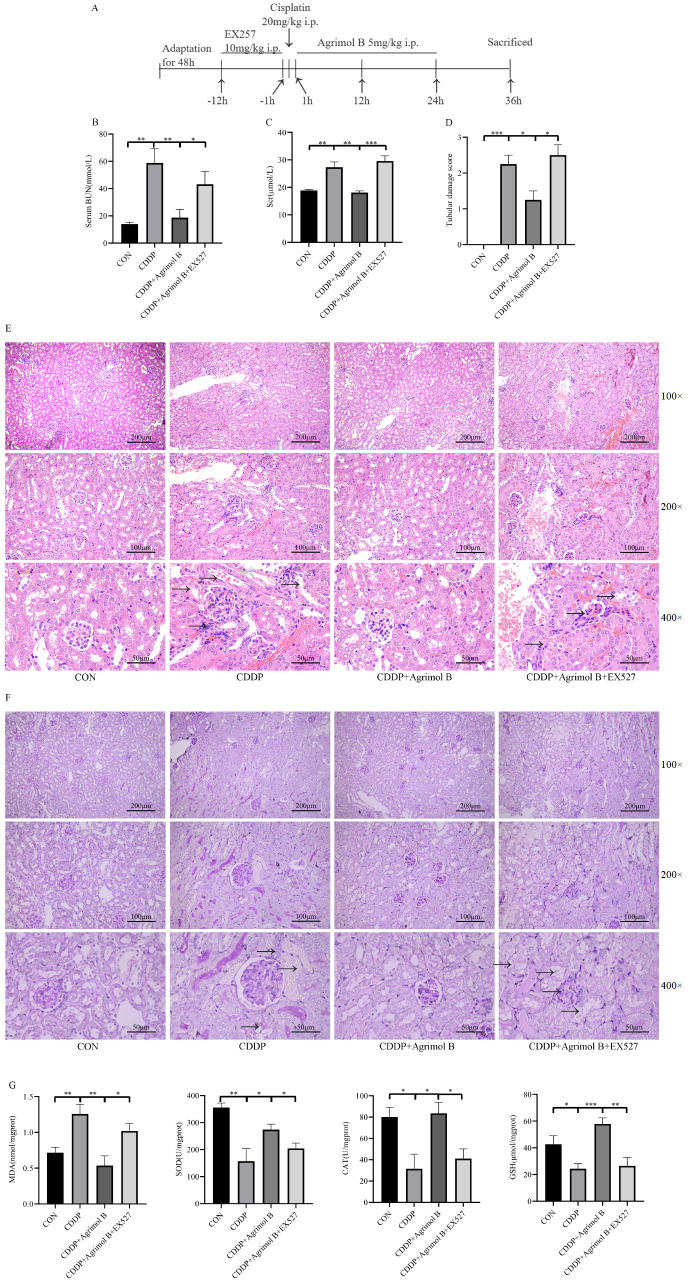
Figure 6 Sirt1 inhibition abrogated the protective effect of agrimol B on CDDP-induced AKI (A) Experimental design. (B) The serum BUN level in each group (n=6 mice per group). (C) The Scr level in each group (n=6 mice per group). (D) Tubular damage score in each group (n=6 mice per group). (E) Kidneys stained with H&E. Representative histological sections were magnified (100×, 200× and 400×) and photographed. (F) Kidneys stained with PAS. Representative histological sections were magnified (100×, 200× and 400×) and photographed. (G) The MDA level, SOD activity, CAT activity and GSH level in each group (n=6 mice per group). All the levels were determined by MDA, SOD, CAT and GSH assays after homogenization of the kidney tissue. Data are presented as the mean±SEM. *P<0.05, **P<0.01, and ***P<0.001.

To investigate the role of Sirt1 in the protective effect of agrimol B on CDDP-induced AKI, we used a specific inhibitor of Sirt1, EX527, to confirm the protective effects of agrimol B. The procedure for establishing the animal model is shown in Figure 6A. As expected, the kidney function indicators BUN and Scr showed that EX527 partially inhibited the renoprotective effect of agrimol B ( Figure 6B,C). Histological staining and scoring revealed that after Sirt1 inhibition, significant destruction of tubular structures was observed (black arrow; Figure 6D‒F); these findings also confirmed that the renoprotective effect of agrimol B was abolished. Subsequently, we examined oxidative stress indicators in kidney tissue and found that the antioxidant effect of agrimol B was significantly inhibited by EX527 ( Figure 6G).

### Agrimol B activates the Nrf2 signaling pathway in a Sirt1-dependent manner in CDDP-induced AKI

**Figure 7:**
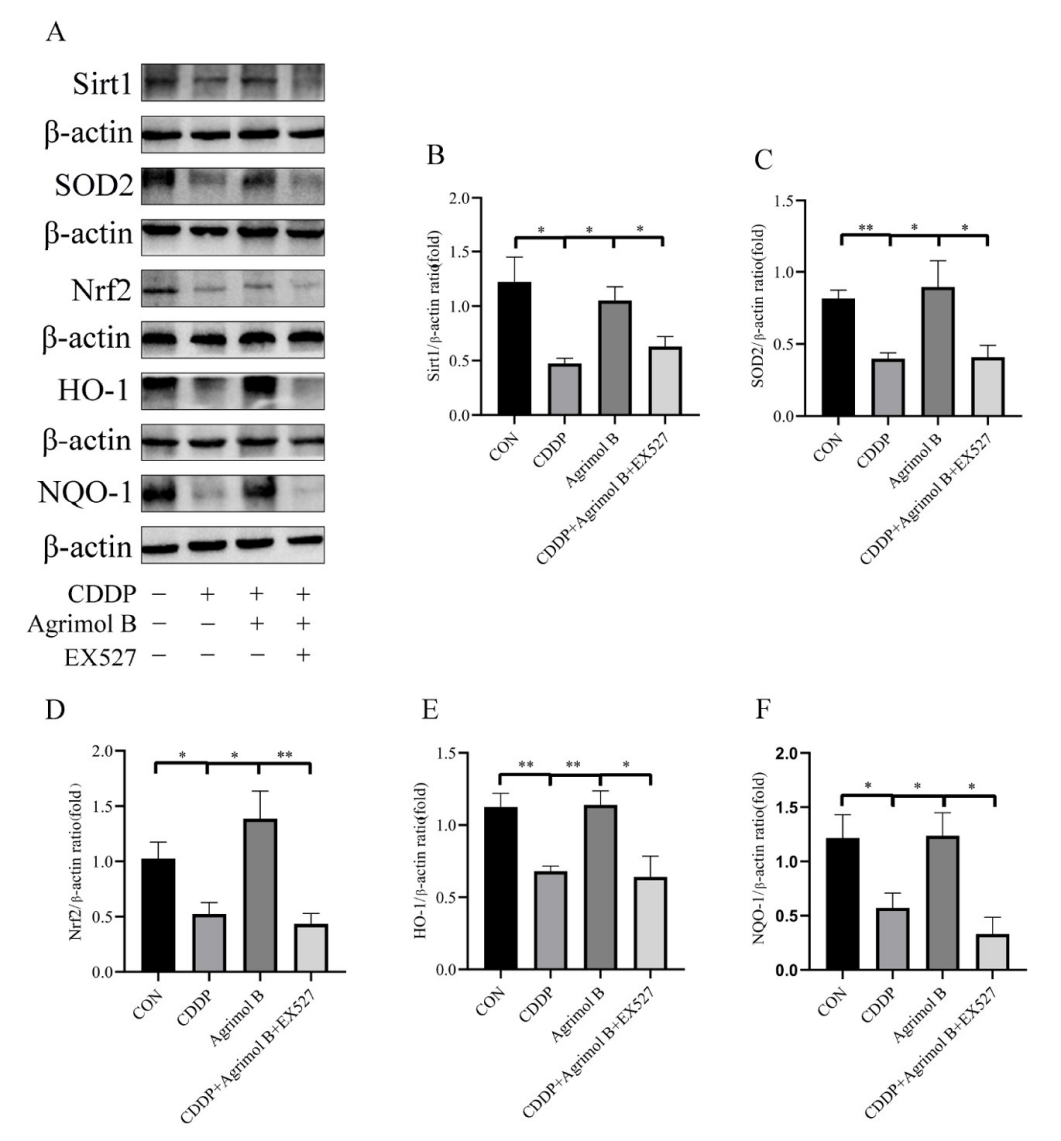
Figure 7 The activation of the Nrf2/HO-1/NQO-1 signaling pathway by agrimol B depended on Sirt1 in CDDP-induced AKI (A) Protein expressions of Sirt1, SOD2, Nrf2, HO-1 and NQO-1. (B) Densitometric analysis of Sirt1 protein expression normalized to that of β-actin. (C) Densitometric analysis of SOD2 protein expression normalized to that of β-actin. (D) Densitometric analysis of Nrf2 protein expression normalized to that of β-actin. (E) Densitometric analysis of HO-1 protein expression normalized to that of β-actin. (F) Densitometric analysis of NQO-1 protein expression normalized to that of β-actin. Data are presented as the mean±SEM. *P<0.05, and **P<0.01.

To further investigate the effect of Sirt1 inhibition on oxidative stress, western blot analysis was performed, and the results showed that EX527 reversed the increase in SOD2 protein expression induced by agrimol B ( Figure 7A,C). Subsequently, we further explored whether Sirt1 is involved in the mechanism linking agrimol B supplementation to the activation of the Nrf2/HO-1/NQO-1 signaling pathway. We examined the expressions of Nrf2 and its downstream proteins following the administration of EX527. The Nrf2 signaling pathway, which was upregulated by agrimol B, was blocked by EX527 ( Figure 7). These results further support the protective effect of agrimol B on CDDP-induced AKI. The Sirt1/Nrf2 signaling pathway improves oxidative damage.

### Agrimol B synergizes with the antitumor activity of CDDP

**Figure 8:**
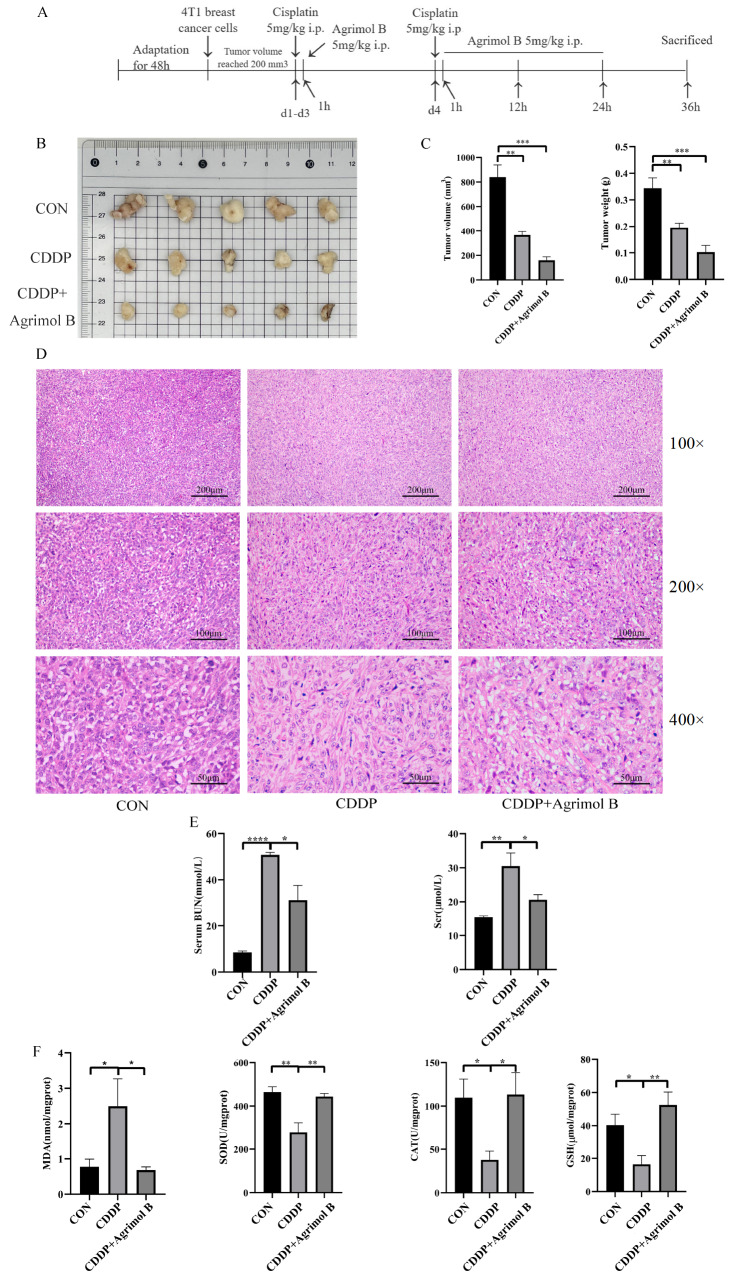
Figure 8 Agrimol B could synergize with the antitumor activity of CDDP (A) Experimental design. (B) Photographs of representative tumor samples. (C) The tumor volume and weight in each group (n=5 mice per group). (D) H&E-stained tumors. Representative histological sections were magnified (100×, 200× and 400×) and photographed. (E) The serum BUN and Scr levels in each group (n=5 mice per group). (F) The MDA concentration, SOD activity, CAT activity and GSH concentration in each group (n=5 mice per group) were determined by MDA, SOD, CAT and GSH assays after homogenization of the kidney tissue. Data are presented as the mean±SEM. *P<0.05, **P<0.01, ***P<0.001 and ****P<0.0001.

Agrimol B was confirmed to attenuate CDDP-induced AKI. However, whether agrimol B can affect the therapeutic effect of CDDP on tumors is still unknown. To address this issue, we further explored a tumor-bearing model ( Figure 8A). The results of the 4T1 xenotransplantation experiment showed that, compared with that in the untreated group, tumor growth was significantly inhibited by CDDP alone. Furthermore, co-treatment with agrimol B had a more potent effect on suppressing tumor growth ( Figure 8B,C). Histopathological examination of the tumor tissues revealed that the tumor cells in the control group were densely packed and closely arranged. These cells exhibited hyperchromatic nuclei and a high nuclear-to-cytoplasmic ratio. CDDP or agrimol B combined with CDDP improved the cell cluster density and increased cell necrosis, resulting in fewer nuclei and less intense staining ( Figure 8D). These observations indicated that CDDP combined with agrimol B inhibited tumor growth. Under tumor-bearing conditions, CDDP treatment induced AKI, as indicated by an increase in the serum BUN and Scr levels ( Figure 8E), which was significantly mitigated by co-treatment with agrimol B. The detection of oxidative stress indicators in the kidney confirmed that agrimol B still played a protective role via its antioxidant effect in the tumor-bearing model ( Figure 8F).

## Discussion

As a commonly used chemical drug, CDDP is widely applied to treat a variety of malignant solid tumors in humans [ 1, 6]. CDDP is metabolized mainly by the kidney, and severe nephrotoxicity limits its maximum efficacy in clinical practice [14]. The most common side effect of CDDP is AKI, for which the mortality rate is 50% [15]. Previous studies have shown that the nephrotoxicity of CDDP may be related to oxidative stress, apoptosis, inflammation, etc. [14]. However, there is still no option for preventing AKI because the underlying mechanism has not been fully elucidated. At present, the most common clinical strategy for managing CDDP-induced AKI is hydration-forced diuresis combined with diuretics or mannitol. However, excessive diuresis can easily lead to dehydration, and patients with poor cardiopulmonary function are also not suitable for excessive fluid volume [ 8, 16, 17]. Other commonly used anti-CDDP renoprotective agents, such as amifostin, can reduce CDDP-induced nephrotoxicity but also cause other serious side effects. Thiols and antioxidants, which have been rated as free radical scavengers in research, can reduce not only CDDP toxicity but also its antitumor effect [ 8, 17, 18]. For patients with severe AKI, renal replacement therapy is often used in the ICU. However, the choice of renal replacement therapy and the timing of initiation and discontinuation are still controversial for different patients and are often related to the occurrence of adverse events related to renal replacement therapy [ 19, 20]. Thus, identifying an effective agent to attenuate CDDP-induced AKI is urgently needed.

In recent years, Chinese herbal medicines based on natural products have attracted much attention due to their minimal side effects and high bioactive potential. *Agrimonia pilosa* Ledeb is a traditional Chinese medicine with various pharmacological activities, including antineoplastic, antioxidative, and anti-inflammatory activities [21]. Agrimoniin, a polyphenolic compound isolated from *Agrimonia pilosa* Ledeb, has been shown to affect intracellular ROS levels and promote apoptosis in cancer cells by regulating the Nrf2 signaling pathway [22]. Agrimol B, which is a polyphenolic compound, serves as the main active ingredient in *Agrimonia pilosa* Ledeb. It was selected as a marker compound for quality control [ 9, 10]. Previous studies have shown that agrimol B can alleviate obesity and related dysfunction by upregulating the expression of the antioxidant protein Sirt1 [10]. Excessive ROS production destroys the redox balance in the kidney and causes oxidative damage, which is a major factor in the pathogenesis of CDDP-induced AKI [23]. Therefore, this study aimed to investigate whether agrimol B could attenuate CDDP-induced AKI by upregulating Sirt1 to protect against oxidative stress without compromising the antitumor effects of CDDP.

In our study, the serum BUN and Scr levels of mice were elevated after CDDP administration, and severe renal histopathological abnormalities, including glomerular injury, tubular dilatation and vacuolation, and tubular epithelial necrosis, were induced; these findings are consistent with previous reports of AKI [ 23, 24]. The concentration of CDDP in renal tubular epithelial cells during metabolism is up to 5 folds greater than that in blood. This high concentration facilitates the mobile transport and passive diffusion of CDDP, resulting in its accumulation in renal proximal tubules. This accumulation leads to injury and cell death, ultimately causing nephrotoxicity [ 24, 25]. After treatment with agrimol B, the serum BUN and Scr levels were significantly reduced, the abnormalities observed via renal histopathology improved, and renal injury was alleviated in mice. This evidence suggested that agrimol B has a protective effect on the kidney and may be used as a potential protective agent against CDDP-induced AKI.

Oxidative stress is one of the most critical factors in CDDP-induced AKI, followed by ROS accumulation [15]. Oxidative stress can promote histopathological lesions by increasing the production of lipid peroxidation products and decreasing the levels of antioxidant enzymes [ 26, 27]. Excessive ROS accumulation can cause oxidation of lipids, proteins and DNA and activate multiple signaling pathways [23]. Our results showed that agrimol B significantly reduced the level of MDA and mitigated the depletion of SOD2, GSH, and CAT, thereby effectively alleviating CDDP-induced oxidative stress, which may be mediated by decreased expression of Sirt1 [28].

Nrf2 is a transcription factor that is widely expressed in a variety of cells. It regulates the inflammatory response, antioxidant defense system, and redox homeostasis to protect cells and organs [ 28‒ 30]. Several studies have shown that Nrf2 exerts a vital protective effect on various models of kidney injury. For example, the activation of Nrf2 can improve age-related kidney injury in mice [28]. *Nrf2* gene deletion significantly reduced renal function and the survival rate in mice with ischemia‒reperfusion (I/R) injury compared to those in wild-type mice [31]. In a streptozotocin-induced diabetic nephropathy model, *Nrf2*-knockout mice also developed more severe kidney injury, which was accompanied by oxidative damage [32]. Sirt1 is an NAD ^+^-dependent deacetylase and is located primarily in the nucleus [10]. Its deacetylation function plays a crucial role in the body, affecting oxidation and inflammation, gene transcription, aging, the cellular stress response, and energy homeostasis [ 29, 33]. Many studies have shown that the activation of Sirt1 can significantly reduce kidney injury. For example, Sirt1 can protect the mouse renal medulla from oxidative damage and plays roles in preventing fibrosis and apoptosis in mouse kidney obstruction [34]. In addition, the activation of Sirt1 can significantly alleviate endoplasmic reticulum stress caused by renal I/R injury in diabetic rats [35]. Increasing evidence suggests that Sirt1 activation may act on Nrf2 through deacetylation [29], thereby activating Nrf2 transcription and expression [36]. Activated Nrf2 binds to antioxidant response elements, regulating the expression of numerous genes that encode antioxidant enzymes such as HO-1, NQO-1, and SOD. This process reduces ROS production, thus exerting a more potent antioxidant effect. Excessive ROS deplete Sirt1 and Nrf2 and disrupt redox homeostasis [ 33, 37, 38]. Previous studies have shown that *Sirt1* knockout in mouse oocytes leads to the downregulation of Nrf2 expression and the exacerbation of oxidative stress [39]. In addition, activation of the Sirt1/Nrf2 signaling pathway has been demonstrated to protect against renal I/R injury in mice and human renal epithelial cell lines via the antioxidant pathway [29]. In our study, treatment with agrimol B promoted the renal protein expressions of Sirt1 and SOD2 and the expressions of endogenous antioxidant systems, including Nrf2, HO-1, and NQO-1, in a mouse model of CDDP-induced AKI. Interestingly, the changes in gene expression were consistent with the changes in protein expression in the kidney. The Sirt1 inhibitor EX527 abolished the protective effect of agrimol B on CDDP-induced AKI, further suggesting that agrimol B activates Nrf2 and its downstream signaling pathways by stimulating Sirt1 expression. Therefore, we concluded that agrimol B ameliorates CDDP-induced AKI by reducing renal oxidative damage through Sirt1/Nrf2 signaling.

Furthermore, agrimol B not only alleviated AKI but also enhanced anti-neoplastic effects when combined with CDDP in a mouse model of breast cancer, increasing its potential for clinical application. However, the mechanism of action of agrimol B in combination with CDDP in antitumor treatment needs further study.

In summary, we used network pharmacology to investigate the potential pharmacological mechanisms of agrimol B in the treatment of CDDP-induced AKI, and multiple targets were predicted. Agrimol B can protect against CDDP-induced AKI by activating the Sirt1/Nrf2 signaling pathway *in vivo*. In addition, agrimol B in combination with CDDP had synergistic antitumor effects on a number of patients, suggesting that this compound is a potential agent for the clinical treatment of CDDP-induced AKI.
